# Comorbidity profiles and inpatient outcomes during hospitalization for heart failure: an analysis of the U.S. Nationwide inpatient sample

**DOI:** 10.1186/1471-2261-14-73

**Published:** 2014-06-05

**Authors:** Christopher S Lee, Christopher V Chien, Julie T Bidwell, Jill M Gelow, Quin E Denfeld, Ruth Masterson Creber, Harleah G Buck, James O Mudd

**Affiliations:** 1 Oregon Health & Science University School of Nursing and Knight Cardiovascular Institute, 3455 SW US Veterans Hospital Road, Portland, OR 97239-2941, USA; 2 Oregon Health & Science University Knight Cardiovascular Institute, 3181 S.W. Sam Jackson Park Rd, Portland, OR 97239, USA; 3 Oregon Health & Science University School of Nursing, 3455 SW US Veterans Hospital Road, Portland, OR 97239-2941, USA; 4 University of Pennsylvania School of Nursing, 418 Curie Blvd., Philadelphia, PA 19104, USA; 5 The Pennsylvania State University College of Nursing, 201 Health and Human Development East University Park, Philadelphia, PA 16802, USA

**Keywords:** Heart failure, Comorbidity, Outcomes, Inpatient

## Abstract

**Background:**

Treatment of heart failure (HF) is particularly complex in the presence of comorbidities. We sought to identify and associate comorbidity profiles with inpatient outcomes during HF hospitalizations.

**Methods:**

Latent mixture modeling was used to identify common profiles of comorbidities during adult hospitalizations for HF from the 2009 Nationwide Inpatient Sample (n = 192,327).

**Results:**

Most discharges were characterized by "common" comorbidities. A "lifestyle" profile was characterized by a high prevalence of uncomplicated diabetes, hypertension, chronic pulmonary disorders and obesity. A "renal" profile had the highest prevalence of renal disease, complicated diabetes, and fluid and electrolyte imbalances. A "neurovascular" profile represented the highest prevalence of cerebrovascular disease, paralysis, myocardial infarction and peripheral vascular disease. Relative to the common profile, the lifestyle profile was associated with a 15% longer length of stay (LOS) and 12% greater cost, the renal profile was associated with a 30% higher risk of death, 27% longer LOS and 24% greater cost, and the neurovascular profile was associated with a 45% higher risk of death, 34% longer LOS and 37% greater cost (all p < 0.001).

**Conclusions:**

Comorbidity profiles are helpful in identifying adults at higher risk of death, longer length of stay, and accumulating greater costs during hospitalizations for HF.

## Background

Management of the heterogeneous disorder of heart failure (HF) is particularly complex in the presence of comorbidities [1-3]. Nearly 60% of adults with HF have five or more chronic comorbidities, a percentage that has increased dramatically over the last twenty years [4]. Both cardiovascular and non-cardiovascular comorbidities observed among adults with HF contribute to condition development, progression and prognosis [5-7]. Comorbidities in HF also vary in etiological association [8], with some conditions like myocardial infarction and peripheral vascular disease having risk factors in common, and others such as cerebrovascular disease and dementia having a direct causal link. Thus, although the influence of specific and/or the number of comorbidities are important to our understanding of HF complexity, identifying naturally occurring patterns among comorbidities may be more helpful in identifying subgroups of HF patients who are more vulnerable to poor outcomes and who would benefit from tailored management strategies [9,10].

Accordingly, the primary aim of this study was to identify and characterize distinct, common, and naturally-occurring profiles among chronic comorbidities in adult hospitalizations for HF. To quantify the clinical relevance of these findings, we also sought to quantify associations among observed comorbidity profiles and the inpatient outcomes of all-cause death, length-of-stay (LOS) and costs.

## Methods

Data from the 2009 Agency for Healthcare Research and Quality (AHRQ) Health Care Utilization Project Nationwide Inpatient Sample (NIS) were used for this study. The NIS is the largest publicly available all-payer inpatient care database in the U.S. The 2009 NIS contains all discharge data from a sampling frame of 4,390 hospitals located in 44 States, representing an approximate 20% stratified sample of U.S. community hospitals. This study was determined to be exempt from ethical review by the Oregon Health & Science University institutional review board because all NIS data are de-identified and includes several other safeguards to protect the privacy of individual patients, physicians and hospitals. This study conforms to the principles of the Declaration of Helsinki. All hospital discharges were examined for the diagnosis of HF by identifying AHRQ clinical classification software [11] diagnosis category 108 (i.e. congestive HF, nonhypertensive (International Classification of Diseases, 9^th^ Edition, Clinical Modifications (ICD-9-CM) codes 398.91, 428.0 428.1 428.20 428.21 428.22 428.23 428.30 428.31 428.32 428.33 428.40 428.41 428.42 428.43 428.9)) [12] and additional ICD-9-CM codes indicative of HF (402.01, 402.11, 402.91, 404.01, 404.03, 404.11, 404.13, 404.91, 404.93) [13,14] as the principal diagnoses. In the NIS, the principal diagnosis was the condition established after study to be chiefly responsible for prompting the hospitalization. Hospitalizations for HF were also verified as being urgent or emergent (i.e. non-elective).

### Assessment of comorbidities

In accordance with a common definition, comorbidities were considered coexisting conditions to HF, the index disorder under study [15]. AHRQ comorbidity software [16] was used to generate binary variables that identify 29 comorbidities in discharge records using ICD-9-CM codes. Seventeen Deyo-Charlson clinical comorbidities [17] were also assessed using ICD-9-CM codes. There was both overlap and additive value in comparing the AHRQ and Deyo-Charlson comorbidities. Hence, a complement of 25 comorbid illnesses from the AHRQ software (acquired immunodeficiency syndrome, alcoholism, deficiency anemia, rheumatoid arthritis/collagen vascular disease, chronic blood loss anemia, chronic pulmonary disease, coagulopathy, depression, diabetes, uncomplicated, diabetes with chronic complications, drug abuse, hypertension, hypothyroidism, lymphoma, fluid and electrolyte disorders, metastatic cancer, other neurological disorders, obesity, paralysis, peripheral vascular disorders, psychoses, pulmonary circulation disorders, solid tumor without metastasis, valvular disease and weight loss) and 7 comorbid illnesses from the Deyo-Charlson comorbidity measures (acute myocardial infarction, cerebrovascular disease, dementia, peptic ulcer disease, mild liver disease, renal disease and moderate/severe liver disease). The number of chronic comorbidities and Charlson Comorbidity Index categories of comorbid burden (low (1–2), medium (3–4), high (5 or more)) [18] were also computed for comparisons.

### Inpatient outcomes

Death was defined in the NIS as in-hospital mortality, and in this paper, is reported as all-cause. Length-of-stay was calculated as the number of nights the patient remained in the hospital for a particular discharge. Length-of-stay in this analysis was all-cause. Total charges reported in the 2009 NIS were converted to costs using hospital-specific cost-to-charge ratios based on actual accounting reports from the Centers for Medicare and Medicaid Services. Costs, in $U.S. 2009, reported in this paper were all-cause.

### Statistical analysis

In accordance with the structure of the NIS database, the primary unit of analysis was hospitalization. Means and proportions with standard errors adjusted for discharge weights were used to describe population estimates derived from this sample. Latent class mixture modeling was used to identify distinct and common comorbidity profiles among binary categorical variables indicating the presence or absence of 32 complementary comorbidities (performed with M*plus* v.6, Los Angeles, California). Latent class mixture model specification was based on procedures explicated by Ram and colleagues [19]. The Lo-Mendell-Rubin adjusted likelihood ratio test (LMRT) [20], convergence (entropy near 1.0), sufficient proportions of the sample in each profile, and posterior probabilities (average probability of belonging in "most likely" profile near 1.0) were used to compare alternative models (e.g. *k* vs. *k*-1 profiles) [21,22]; discharge weights were incorporated into all latent class models. Observed profiles were labeled according to dominant and differentiating comorbidities. Differences in comorbidities among observed profiles were quantified using χ^2^ tests. Multinomial logistic regression models were generated to calculate marginal probabilities (range -1 to 1, sum to zero) [23] of belonging to each profile based on single comorbid conditions.

Generalized linear modeling was used to quantify associations among observed comorbidity profiles and inpatient death (relative risk (RR)) length-of-stay (incident rate ratio (IRR) based on the negative binomial distribution corrected for over-dispersion) and inpatient costs (gamma distribution for relative differences, and Duan’s smearing retransformation of log-transformed cost [24] to estimate absolute differences in raw 2009 U.S. dollars) (performed with Stata v.11MP, College Station, Texas). Associations between the number of comorbidities and Charlson categories of comorbid burden and inpatient outcomes also were generated for context and comparison. Discharge weights were applied in all generalized linear models, and all estimates were adjusted for age, gender, race, median income of the patient’s zip code, primary expected healthcare payer, and weekend vs. weekday admissions, as well as hospital bed size (small, medium, or large) control (government or private, government/nonfederal (public), private/non-profit, private/investor owned, private), location (rural, urban), region (Northeast, Midwest, South, West) and teaching status (non-teaching, teaching).

## Results

### Characteristics of the population

The sample consisted of 192,327 hospitalizations (population estimate = 976,664) with HF listed as the principal diagnosis (Table 1). Approximately 51% of discharges were for women, and the average age was almost 73 years. Death occurred during just over 3% of hospitalizations, the average length-of-stay was 5.2 days (median = 4 days), and the average inpatient cost was $11,313 (Table 2). In total, there were 12,966 inpatient deaths, 2.2 million inpatient days, and $4.6 billion in inpatients costs translating to population estimates of 30,798 deaths, 5.1 million inpatient days, and a total of $10.3 billion in inpatient costs.

**Table 1:** Characteristics of the sample (n = 192,327^*^)

**Discharge and hospital characteristics**	**Estimate (mean or proportion)**	**95% CI**
Age (in years)	72.88	(72.82-72.95)
Gender (% female)	50.77%	(50.55%-51.00%)
Race/Ethnicity^†^		
White	68.02%	(67.79%-68.24%)
Black	18.98%	(18.79%-19.17%)
Hispanic	7.73%	(7.61%-7.86%)
Asian or Pacific Islander	1.79%	(1.72%-1.85%)
Native American	0.54%	(0.51%-0.58%)
Other	0.30%	(0.29%-0.30%)
Median zip code income national quartile		
First	31.60%	(31.39%-31.81%)
Second	27.00%	(26.80%-27.21%)
Third	22.63%	(22.44%-22.82%)
Fourth	18.77%	(18.59%-18.95%)
Primary healthcare payer		
Medicare	74.31%	(74.11%-74.51%)
Medicaid	7.99%	(7.87%-8.11%)
Private insurance	12.24%	(12.09%-12.39%)
Self-pay	3.54%	(3.46%-3.63%)
No charge	0.32%	(0.30%-0.36%)
Other	1.59%	(1.54%-1.65%)
Admission on a weekend	23.42%	(23.23%-23.60%)
Hospital bed size		
Small	13.28%	(13.12%-13.43%)
Medium	23.88%	(23.68%-24.07%)
Large	62.85%	(62.63%-63.07%)
Control		
Government or private	59.53%	(59.31%-59.75%)
Government, nonfederal	7.18%	(7.07%-7.30%)
Private, non-profit	18.37%	(18.20%-18.55%)
Private, investor owned	10.72%	(10.58%-10.86%)
Private	4.19%	(4.10%-4.28%)
Location		
Rural	14.92%	(14.76%-15.08%)
Urban	85.08%	(84.92%-85.23%)
Region		
Northeast	22.35%	(22.16%-22.54%)
Midwest	23.40%	(23.21%-23.59%)
South	39.37%	(39.15%-39.59%)
West	14.87%	(14.72%-15.03%)
Teaching status		
Non-teaching	58.78%	(58.56%-59.00%)
Teaching	41.22%	(41.00%-41.44%)

^*^Estimates generated using discharge weights (population estimate n = 976,664).

^†^data missing for 14.3% of discharges. CI, confidence interval.

**Table 2:** Comorbidities and outcome characteristics (n = 192,327^*^)

**Comorbidities and outcomes**	**Estimate (mean or proportion)**	**95% CI**
Number of comorbidities	7.54	(7.53-7.55)
Charlson comorbidity category		
Low (1–2)	0.29%	(0.26%-0.31%)
Medium (3–4)	17.29%	(17.12%-17.46%)
High (5+)	82.42%	(82.25%-82.60%)
Inpatient death	3.15%	(3.08%-3.23%)
Inpatient length of stay (days)	5.22	(5.20-5.25)
Inpatient cost (2009 $US)	$11,313.28	($11,233.77-$11,329.80)

^*^Estimates generated using discharge weights (population estimate n = 976,664).

*Abbreviations:* CI, confidence interval.

### Number of comorbid illnesses, Charlson categories and inpatient outcomes

**Figure 1:**
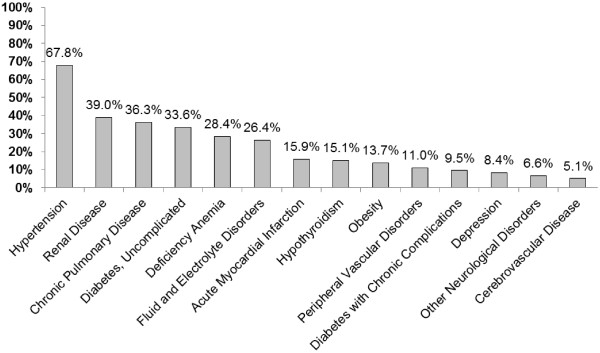
**Prevalence of comorbidities in adult hospitalizations with a principal diagnosis of heart failure.** Prevalence estimates were generated using discharge weights (n = 192,327; population estimate n = 976,664). Only conditions with a prevalence of 5% or greater are displayed for economy of presentation.

The most prevalent comorbidities in this sample were hypertension, renal disease, chronic pulmonary disease, uncomplicated diabetes mellitus, and deficiency anemia (Figure 1). The average number of comorbid illnesses was 7.5, and the vast majority of discharges (82.4%) were for patients with a high Charlson comorbidity category (Table 2). There was a relatively small, but positive influence of the number of comorbidities on the risk of inpatient death (RR = 1.03, 95% confidence interval (CI) =1.02 -1.04), length-of-stay (IRR = 1.05, 95% CI =1.04-1.05), and inpatient cost (relative increase = 5.4%, 95% CI =5.1%-5.6%) (all p < 0.0001). Only 0.3% of discharges were for adults with a low Charlson comorbidity category – a subgroup too small for effective comparisons. Discharges for adults with a high vs. a medium Charlson comorbidity category, however, were associated with a greater risk of inpatient death (RR = 1.16 (95% CI = 1.08-1.25), longer length of stay (IRR = 1.15 (95% CI = 1.13-1.17), and greater inpatient cost (relative increase = 12.5%, 95% CI =10.3%-14.7%) (all p < 0.0001).

### Identifying profiles of comorbidity

**Figure 2:**
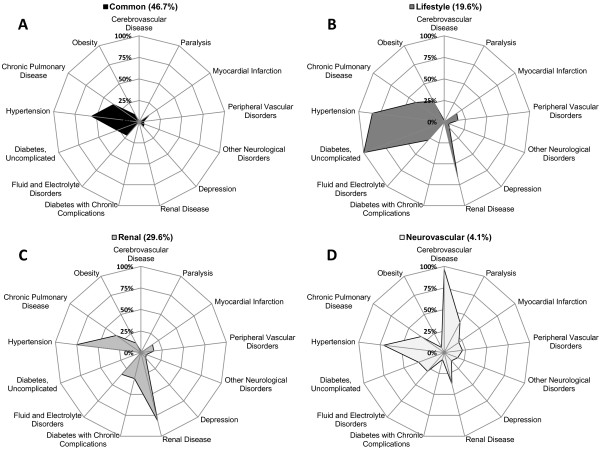
**Four observed comorbidity profiles in adult hospitalizations with a principal diagnosis of heart failure.** Radar graphs present within-profile prevalence of differentiating comorbidities from 0% (center of graph) to 100% (outside of graph). The common profile **(A)** had the lowest prevalence of cerebrovascular disease, myocardial infarction, peripheral vascular disorders, depression, renal disease, fluid and electrolyte disorders, hypertension, and obesity compared with the other profiles. Key attributes of the lifestyle profile **(B)** were high rates of uncomplicated diabetes, hypertension, chronic pulmonary disorders and obesity. Key attributes of the renal profile **(C)** were high rates of renal disease, diabetes with complications and fluid and electrolyte imbalances. Key attributes of the neurovascular profile **(D)** were high rates of cerebrovascular disease, paralysis, myocardial infarction, peripheral vascular disease, neurological disorders and depression. Prevalence estimates were generated using discharge weights (n = 192,327; population estimate n = 976,664). All differences by comorbidity profile were statistically significant (all p < 0.0001).

Among the 32 conditions considered, four distinct comorbidity profiles were identified (model entropy = 0.71; LMRT = 16,296; p < 1×10^-7^). The largest profile (n = 89,518 (46.7%), Figure 2-A), which was labeled as "common," included HF discharges with relatively few, but common comorbidities. There were 2 fewer comorbidities on average in the common profile than the other profiles. The common profile also had the lowest prevalence of cerebrovascular disease (0.8%), myocardial infarction (13.9%), peripheral vascular disorders (5.2%), depression (7.5%), renal disease (0.5%), fluid and electrolyte disorders (20.8%), hypertension (55.8%), and obesity (9.3%) compared with the other profiles. The prevalence of uncomplicated diabetes in the common profile was 27.4% and there were no cases of diabetes with chronic complications. The common profile, named in part because of the size of the profile and the relatively limited comorbid burden, served as a reference to which the other profiles could be compared.

A "lifestyle" profile (n = 37,797 (19.6%), Figure 2-B) was characterized by a greater percentage of HF discharges with uncomplicated diabetes, hypertension, chronic pulmonary disorders and obesity than any other profile (all p < 1×10^-7^). A "renal" profile (n = 57,228 (29.6%), Figure 2-C) had a greater percentage of HF discharges with renal disease, diabetes with complications and fluid and electrolyte imbalances than any other profile (all p < 1x10^-7^). Finally, a "neurovascular" profile (n = 7,784 (4.1%), Figure 2-D) had a greater percentage of HF hospitalizations with cerebrovascular disease, paralysis, myocardial infarction, peripheral vascular disease, neurological disorders and depression than any other profile (all p < 1×10^-7^).

Key profile-differentiating comorbidities are presented in Table 3. In the presence of paralysis, the probability of belonging to the neurovascular comorbidity profile is 91.1% and the probability of belonging to the other profiles is dramatically reduced. Similarly, the presence of cerebrovascular disease is associated with a high probability (76.5%) of belonging to the neurovascular profile and reduced probability of belonging to the other comorbidity profiles. The presence of diabetes with complications (73.8%) and renal disease (53.3%) increases the probability of belonging to the renal profile, and the presence of uncomplicated diabetes (57.3%) and obesity (22.7%) increase the probability of having the lifestyle profile of comorbidities.

**Table 3:** Key differentiating comorbidities: marginal probabilities (n = 192,327*)

**Comorbidity**	**Common n = 89,518**^**† **^**(46.7%)**	**Lifestyle n = 37,797**^**† **^**(19.6%)**	**Renal n = 57,228**^**† **^**(29.6%)**	**Neurovascular n = 7,784**^**† **^**(4.1%)**
Paralysis	-0.422	-0.187	-0.302	**0.911**
Cerebrovascular disease	-0.417	-0.129	-0.220	**0.765**
Diabetes with complications	-0.515	-0.217	**0.738**	-0.006
Renal disease	-0.755	0.224	**0.533**	-0.002
Diabetes, uncomplicated	-0.142	**0.573**	-0.428	-0.003
Obesity	-0.175	**0.227**	-0.028	-0.024

All marginal probabilities were statistically significant (all p < 0.0001). Marginal effects in probability scale sum to zero for each comorbidity, with values closest to ±1.0 indicating the strongest differentiation.

^*^Estimates generated using discharge weights (population estimate n = 976,664).

^†^Population estimates for totals of discharges fitting the common, lifestyle, renal and neurovascular comorbid illness profiles were 455851, 191595, 289613, and 39604, respectively.

### Profiles of comorbidity and inpatient outcomes

Associations among observed comorbidity profiles and inpatient outcomes are presented in Table 4. Relative to the common comorbidity profile, inpatient death was 30.2% more likely for HF discharges fitting renal profile and 44.5% more likely for HF discharges fitting the neurovascular profile (both p < 1×10^-7^). Relative to the common comorbidity profile, discharges within the lifestyle profile were associated with a 14.9% longer length of stay, discharges within the renal profile were associated with a 26.9% longer length of stay, and HF discharges fitting the neurovascular profile were associated with a 33.6% longer length of stay (all p < 1×10^-7^). In raw days of length-of-stay, hospitalizations for patients fitting the lifestyle comorbidity profile were 0.68 days longer, hospitalizations for patients fitting the renal profile were 1.26 days longer, and hospitalizations for patients fitting the neurovascular profile were 1.57 days longer on average than HF discharges fitting the common profile (all p < 1×10^-7^). Relative to the common comorbidity profile, discharges within the lifestyle profile were associated with 11.9% greater inpatient costs, those fitting the renal profile were associated with 23.5% greater inpatient costs, and those within the neurovascular profile were associated with 36.7% greater inpatient costs (all p < 1×10^-7^). In raw 2009 US dollars, hospitalizations for patients fitting the lifestyle profile cost $1,422.36 more, hospitalizations for patients fitting the renal profile cost $2,199.26 more, and hospitalizations for patients fitting the neurovascular profile cost $2,809.39 more on average compared with HF discharges fitting the common profile.

**Table 4:** Adjusted differences in inpatient outcomes by comorbidity profile (n = 192,327^*^)

	**Inpatient death**		**Length-of-stay**		**Inpatient cost**	
**Comorbidity profile**	**RR (95% CI)**	**p-value**	**IRR (95% CI)**	**p-value**	**Relative cost (95% CI)**	**p-value**
Lifestyle^†^	1.023 (0.945-1.108)	0.573	1.149 (1.135-1.163)	<0.001	111.9% (110.0%-113.9%)	<0.001
Renal^†^	1.302 (1.223-1.387)	<0.001	1.269 (1.254-1.284)	<0.001	123.5% (121.5%-125.4%)	<0.001
Neurovascular^†^	1.445 (1.280-1.631)	<0.001	1.336 (1.291-1.381)	<0.001	136.7% (131.3%-142.3%)	<0.001

*Note*: All estimates are adjusted for age, gender, race, median income of the patient’s zip code, primary healthcare payer, and weekend vs. weekday admissions, as well as hospital bed size (small, medium, or large) control (government or private, government/nonfederal (public), private/non-profit, private/investor owned, private), location (rural, urban), region (Northeast, Midwest, South, West) and teaching status (non-teaching, teaching).

^*^Estimates generated using discharge weights (population estimate n = 976,664).

^†^Relative to the "common" comorbidity profile.

*Abbreviations*: CI, confidence interval calculated from weighted standard errors; IRR, incident rate ratio; RR, relative risk.

## Discussion

Management of HF is complicated by prevalent cardiovascular and non-cardiovascular comorbidities [25]. In this nationally-representative inpatient sample of 192,327 adult hospitalizations with a principal diagnosis of HF, there was an average of 7.5 comorbidities and the vast majority of hospitalizations (82.4%) were categorized as having a high Charlson comorbid burden. The main finding of this study is the identification of four distinct and naturally occurring comorbidity profiles that were associated with significant differences in the all-cause inpatient outcomes of death, length-of-stay and cost. Relative to the 46.7% of hospitalizations with a "common" comorbidity profile, the 19.6% of HF hospitalizations fitting a "lifestyle" profile had longer length-of-stay and greater costs, and the 29.6% of HF hospitalizations within a "renal" comorbidity profile and the 4.1% of HF hospitalizations with a "neurovascular" comorbidity profile were associated with higher risk of inpatient death, longer length-of-stay and greater costs.

Similar to the findings of other studies, we observed a direct relationship between the number of comorbidities [26-28] as well as categories of Charlson comorbid burden [29] and clinical outcomes among adults with HF. Specifically, we observed an adjusted 3%-5% increase in the risk of death, extension in length-of-stay and accrual of inpatient costs for additional comorbidities. The number of comorbidities and the proportion of discharges associated with 5 or more comorbidities in this sample are greater than prior reports in HF [4,28], which can be explained, at least in part, by our inclusion of 32 comorbidities that were of both cardiovascular and non-cardiovascular origin. An extreme minority of hospitalizations in this sample involved patients with a low comorbid burden; thus, we compared hospitalizations fitting a moderate and high Charlson comorbid risk. High Charlson comorbid burden was associated with 12%-16% increase in the risk of death, extension in length-of-stay and accrual of inpatient costs compared with hospitalizations for adults with a moderate comorbid burden. In sum, our findings provide further evidence in support of both the number of comorbidities and categorization of comorbid burden as having a significant influence on inpatient outcomes in HF.

There is elegance in the simplicity of counting comorbidities, considering thresholds that represent complex comorbidity (i.e. 5 or more), and weighing counts of comorbidities based on established associations with 1-year mortality (i.e. Charlson). A major limitation to these approaches to comorbidities in HF, however, is the inability to consider how some comorbidities are naturally independent, others have common risk factors and/or pathogenic pathways, and others still have direct causation [8]. Moreover, single comorbidity inventories often omit key conditions that are highly-prevalent in HF (e.g. the Charlson excludes hypertension, and the AHRQ comorbidities do not include myocardial infarction), and ineffectively capture condition severity (e.g. the Charlson does not differentiate diabetes with and without complications, and the AHRQ comorbidities do not differentiate mild from moderate to severe liver disease). Thus, our finding of four distinct, naturally-occurring profiles among comorbidities in adult HF hospitalizations builds upon prior research that has involved single comorbidity inventories or considered all comorbidities as being etiologically and statistically independent.

The largest comorbidity profile we observed involved the fewest and common comorbidities. In fact, the only differentiating factors to identity patients fitting this profile were prevalent HF comorbidities (e.g. hypertension, chronic pulmonary disease, and uncomplicated diabetes) and the absence of renal disease, which had an overall prevalence of 39% but only affected 0.5% of discharges in this profile. Based on this sample, there is an estimated 455,851 hospitalizations in the U.S. for HF patients fitting this comorbidity profile annually, and they experience the most favorable inpatient outcomes. The next hazardous profile we observed was differentiated by comorbidities that are linked to lifestyle, including 100% prevalence of uncomplicated diabetes, 84% prevalence of hypertension, 40% prevalence of chronic pulmonary disease, and 27% prevalence of obesity. The triad of diabetes, hypertension and obesity is well-recognized as integral characteristics of metabolic syndrome [30], and majority of chronic pulmonary disease is thought to be attributable to cigarette smoking [31]. There is an estimated 191,595 annual hospitalizations for HF patients fitting this lifestyle comorbidity profile that is associated with longer length-of-stay and greater inpatient cost. Fitting the lifestyle profile on discharges for HF should serve as a red flag for greater healthcare utilization, and interventions to reduce the risk of poor inpatient outcomes for this profile should be tailored to improving lifestyle in general in addition to treating specific comorbidities. Since hypertension and diabetes mellitus were prominent features of both the common and lifestyle comorbidity profiles, dietary modifications and aerobic exercise are two specific lifestyle recommendations that may be helpful in the reduction of cardiovascular risk in these two groups [32]. It is also known that hypertension and obesity are more prevalent among HF patients with preserved versus reduced ejection fraction [33]; thus, interventions to reduce the risk of poor clinical outcomes may be further tailored by HF type within the observed comorbidity profiles.

The next hazardous profile we observed was centered on a high prevalence of renal disease (82%) and diabetes with complications (31%). The combination of renal disease and diabetes has been shown by others to be associated greater risk of death and hospitalization in HF [34]. Over 3/4 of HF discharges within this renal profile also had hypertension and slightly more than 1/3 had fluid and electrolyte disorders. Diabetes and hypertension are widely recognized as the leading causes of renal disease [35], and cardiorenal syndrome [36] is a challenging condition to manage particularly during acute hospitalization for HF [37]. Given the nature of these data, it is not possible to understand the temporal and/or causal nature of relationships among the key differentiating comorbidities in the renal or other profiles. But, HF hospitalizations for patients fitting this profile were associated with a 30% increase in the risk of inpatient death and 27% and 24% increases in length-of-stay and inpatient costs, respectively and there is an estimated 289,613 annual HF discharges in the U.S. for patients fitting this comorbidity profile.

The most hazardous comorbidity profile identified was differentiated from the others by extremely high rates of cerebrovascular disease, and the highest prevalence of paralysis, myocardial infarction, peripheral vascular disorders, neurologic disorders and depression. Peripheral vascular disease is often paired with comorbid cerebrovascular disease [38], and is a determinant of worse outcomes in patients with myocardial infarction [39]. There also is a link between both cerebrovascular disease [40] and myocardial infarction [41] and depression. Thus, end-organ damage of global vascular disease appeared to be central to this profile. Based on this sample there are more than 39,000 HF hospitalizations for patients fitting this neurovascular comorbidity profile in the U.S. annually; there was a 45% increase in the risk of inpatient death, 34% increase in length-of-stay and 37% increase in inpatient cost associated with discharges for HF patients fitting this profile. Patients fitting the renal or neurovascular profiles upon admission for HF should be assumed to require greater healthcare utilization and should be enrolled in disease management and care transition programs to mitigate the overall impact on global healthcare resources and improve patients’ quality-of-life.

There are several existing schemes for classifying the risk of poor inpatient outcomes in HF. For example, Fonarow and colleagues determined that blood urea nitrogen, admission systolic blood pressure, and serum creatinine were predictive of inpatient mortality among adults admitted for decompensated HF [42]. Coronary artery disease, renal insufficiency and diabetes were more prevalent in the high- versus the low-risk groups in that study. As an additional example, Peterson and group determined that age, systolic blood pressure, blood urea nitrogen, heart rate, serum sodium, coexisting chronic obstructive pulmonary disease, and race were predictive of inpatient mortality among adults admitted for decompensated HF [43]. Thus, the incorporation of coexisting comorbidities into inpatient prognostic models is not new. But, what our findings add to extant knowledge about the influence of comorbidities on inpatient outcomes is a new approach the naturally-occurring clusters of prevalent and coexisting that complicate HF admissions and are associated with significant differences in inpatient outcomes.

### Strengths and limitations

The present study has strengths and limitations. Using the NIS as a nationally-representative sample of patients admitted for the principal diagnosis of HF and applying sampling weights allows us to generalize estimates for comorbidity prevalence, composition of comorbidity profiles and inpatient outcomes that are generalizable to a much larger population than the sample studied. Tradeoffs for this strength are the limitations of using hospitalization (and not patient) as the unit of analysis, and that national representativeness does not translate to generalizability to all persons with HF. There are also challenges to using ICD-9-CM codes for the identification of HF and comorbidities alike. The ICD-9-CM codes chosen for this study, however, are highly predictive of HF when compared with clinical criteria [12], and are established codes for performance measures in HF [13]. Like most research involving administrative databases, there may have been under-reporting of chronic comorbidities in the NIS [44]. It is also likely that the comorbidity profiles we identified are dynamic rather than fixed; thus, these profiles are likely to evolve over time along with the progression of both HF and concomitant comorbidities. We are also unable to distinguish casual relationships among comorbidities, those simple having risk factors in common and the potential pathological links to HF (e.g. complication of, predisposing factor for, or unrelated) in this single evaluation of administrative data; hence, that was not our goal.

We also did not differentiate between HF with preserved or unpreserved ejection fraction. There are important socio-demographic (e.g. older age and female gender), etiological (e.g. greater prevalence of hypertension and atrial fibrillation, and less frequent coronary artery disease) [45], and non-cardiovascular comorbidity differences (e.g. high prevalence of chronic lung, liver and gastric disorders) comparing HF patients with preserved versus reduced ejection [46]. Hence, it will be important to differentiate comorbidity profiles by groups of HF patients with preserved and reduced ejection fraction in future research. Because of the administrative nature of this data source, we were unable to incorporate granular clinical data (e.g. ejection fraction, which ventricles are involved, blood pressure, glomerular filtration rate, and functional performance measures). Hence, future research is warranted to examine the influence of comorbidity profiles along with clinical data including, but not limited to, a) the assessment of HF with preserved versus reduced ejection fraction, b) the natural history of comorbidities over time and how they relate to the evolution of HF, c) different manifestations of HF (e.g. left, right, or bi-ventricular dysfunction), and d) other prognostic clinical data.

## Conclusion

Among a large sample of adult hospitalizations for HF, we observed four distinct comorbidity profiles that were associated with large and significant differences in the risk of inpatient death, length-of-stay and inpatient cost. Naturally-occurring patterns of cardiovascular and non-cardiovascular comorbidities, not simply the number of comorbidities, should be the focus of future research and the target of future interventions aimed at reducing the risk of inpatient death and healthcare utilization.

## Abbreviations

AHRQ: Agency for Healthcare Research and Quality; CI: Confidence interval; HF: Heart failure; ICD-9-CM: International Classification of Diseases, 9^th^ Edition, Clinical Modifications; IRR: Incident rate ratio; LMRT: Lo-Mendell-Rubin adjusted likelihood ratio test; LOS: Length of stay; NIS: Nationwide inpatient sample; RR: Relative risk.

## Competing interests

The authors declare that they have no competing interests.

## Authors’ contributions

CSL conceived of the study, gained access to these data, performed all statistical analysis and led the development of the manuscript. CC, JMG, and JOM served as cardiovascular medicine experts, made substantive improvements to the analysis and written manuscript, and took the lead on describing the clinical relevance of these findings. HGB served as an external expert on the comorbidities in heart failure and provided substantive feedback on the analysis and made major contributions to the writing of the manuscript. JTB, QED, and RMC were integrally involved with the statistical analysis and assisted with final model solutions and presentation of these data; they also made substantive contributions to the writing of the manuscript. All authors read and approved the final manuscript.

## Pre-publication history

The pre-publication history for this paper can be accessed here:

http://www.biomedcentral.com/1471-2261/14/73/prepub
